# Comments on “A Shorter Door-in-Door-out Time Is Associated with Improved Outcome in Large Vessel Occlusion Stroke”

**DOI:** 10.5811/westjem.18668

**Published:** 2024-06-28

**Authors:** Gillian Cooper, Vainavi Gambhir, Zoe Gasparotti, Samantha Camp, William Gum, Robinson Okolo, Riya Raikar, Chad Schrier, Jessica Downing, Quincy K. Tran

**Affiliations:** * University of Maryland School of Medicine, Department of Emergency Medicine, Baltimore, Maryland; † University of Maryland School of Medicine, The R Adams Cowley Shock Trauma Center, Baltimore, Maryland; ‡ University of Maryland School of Medicine, Department of Emergency Medicine, The Research Associate Program in Emergency Medicine and Critical Care, Baltimore, Maryland; § University of Maryland School of Medicine, Baltimore, Maryland; ∥ University of Maryland Medical Center, The Critical Care Resuscitation Unit, Baltimore, Maryland; ¶ University of Maryland Medical Center, Department of Stroke Neurology, Baltimore, Maryland

Dear Editors:

We read with great interest, “A Shorter Door-in-Door-out Time Is Associated with Improved Outcome in Large Vessel Occlusion Stroke,” by Sigal et al.^1^ Firstly, we would like to congratulate the authors on their *WestJEM* publication that highlights important concepts for management of patients with ischemic stroke from large vessel occlusion (LVOS) in the emergency department (ED). However, we had a few comments regarding their conclusions about door-in-door-out (DIDO) time and stroke outcomes. Notably, the title states that shorter DIDO time correlates to improved stroke outcomes, but their multivariable logistic regression analysis demonstrated that DIDO was not statistically significant (Table 4, OR 1.13, 95% CI 0.99–1.30). While the univariate analysis suggested that ED patients with good outcome had shortened DIDO, this conclusion should not be made when their multivariate regression suggested otherwise.

The authors’ findings and discussion also suggest that ED DIDO times are not relevant to stroke outcomes. This is controversial because there were also conflicting reports from previous studies.^2^ ^,^ ^3^ In this present paper, the authors looked at specific time intervals for DIDO thresholds (≤60 minutes, >60 minutes, ≤90 minutes, >90 minutes, ≤120 minutes, >120 minutes), which they included in their univariate analysis but not their multivariable logistic regression analysis. By categorizing continuous variables, the authors risked losing the granularity of the variables and perhaps statistical power; however, it is unknown whether these intervals would have shown significance on multivariable logistic regression analysis, as opposed to assessing DIDO solely as a continuous variable in the logistic regression.

On the other hand, the authors reported that they calculated 90-day modified Rankin Scale (mRS) via electronic health records (EHR) and also retrospectively calculated 90-day mRS values for 77 (18%) of their patients. However, retrospective calculations of mRS are inherently biased, as clinicians tend to score a patient’s disability as higher than what was experienced by the patient, and their quality of life lower.^4^ ^,^ ^5^ Therefore, it is possible that outcome measures were more subjective and could affect the authors’ analyses.

Additionally, data from our comprehensive stroke center (CSC), for which patients with LVOS and thrombectomy undergo prospective 90-day mRS assessment as part of the clinical care for all stroke patients, reported that different time intervals are associated with improved outcome for patients with LVOS. We analyzed the data of 203 patients with LVOS who presented to our CSC via our critical care resuscitation unit (CCRU) between January 2019–May 2021 for thrombectomy, using a machine learning algorithm (classification and regression tree [CART]). The CART algorithm uses recursive partitioning to identify important predictors and assign these predictors with relative variable importance (RVI). The most important predictors would be given a RVI of 100%; other predictors would subsequently be assigned RVI values as percentage of the most important factor.

Similarly to Sigal et al’s study, our analysis found that while National Institutes of Health Stroke Scale and age were significant factors associated with patient outcomes (Appendix 1), the ED DIDO time was also a significant factor with a RVI of 27.6% (Appendix 1). Furthermore, the DIDO time at our CSC’s resuscitation unit was also an important factor with a RVI of 62.7%. All the time intervals in our analysis were entered in the CART as continuous variables. Nevertheless, we hypothesize that the lack of significance in the authors’ multivariable logistic regression may be because their patients’ 90-day mRS values were obtained via EHR, in contrast to our data, which resulted from prospectively collected 90-day mRS values. However, the result that the CCRU’s DIDO was a significant factor with higher RVI values than ED DIDO for patients’ outcome could also provide another possible explanation for the findings in Sigal et al and Scheving et al that ED DIDO might not be a significant factor. It could be that what was delayed in ED DIDO time was later made up by the teams at the CSC once patients were transferred from the EDs. However, further studies are necessary to confirm or refute our observation.

Despite our concerns, we wholeheartedly agree with the authors that clinicians need to expedite patients who have LVOS to undergo thrombectomy, regardless of where they are during the critical time period.

## Supplementary Information



