# Supplementary material for: Comments on “A Shorter Door-in-Door-out Time Is Associated with Improved Outcome in Large Vessel Occlusion Stroke”
Source: West J Emerg Med. 2024 Jun 28;25(5):856–7. doi: 10.5811/westjem.18668 (PMC11418866; doi:10.5811/westjem.18668)
Supplement: Supplementary file 1 [file wjem-25-856-s001.docx]

**Appendix 1**

**Table 1**

| Lactate Range mmol/L | Patients in Cohort | Patients with Outcome | Survival Probability | Survival Percentage |
| --- | --- | --- | --- | --- |
| 0-2 | 270990 | 2860 | 0.9894 | 98.94% |
| 2-4 | 92059 | 3151 | 0.9658 | 96.58% |
| 4-6 | 16004 | 2131 | 0.8669 | 86.69% |
| 6-8 | 6079 | 1667 | 0.7258 | 72.58% |
| 8-10 | 3506 | 1337 | 0.6187 | 61.87% |
| 10-12 | 2394 | 1029 | 0.4298 | 42.98% |
